# Letter from Dr. Meigs

**Published:** 1843-01-21

**Authors:** Ch. D. Meigs

**Affiliations:** Philadelphia


*Philadelphia, Jan.* 16, 1843.

To the Editor of the Examiner.

Dear Sir,—You are aware, that in the course of the last winter, there was much conversation in the medical circles upon the subject of puerperal fever, which at the time was committing some ravages in our city, and filling the air with rumours of its very extensive and pernicious prevalence.

It happened that Dr. Rutter, the author of the letter which I have the honour now to transmit to you, who at that juncture was engaged in a most extensive midwifery practice, had the unhappiness to encounter a very considerable number of the cases; a far greater number, in fact, than fell under the notice of any other practitioner. Indeed, I believe that his practice in that department of the profession, was greater than that of any other gentlemen, which was probably the cause of his seeing a greater number of the cases.

You will perceive from the letter itself, that Dr. R. has encountered full seventy cases of the malady, of which only some fourteen or fifteen cases proved fatal. As it ever must be distressing to any sensitive mind to fail in curing the disease of a patient confided to one's science, skill, or attention, it is easy to imagine how poignant will be the feelings of such a mind, when fourteen or fifteen females are lost under circumstances connected with their lying-in. Doubtless no man could more sensibly feel such distress than the author of the letter in question; and yet, I think, you will agree with me, that upon a review of his connection with that distressing epidemic, he has abundant causes why he should be congratulated upon a success in the treatment of it, not exceeded by any one, so far as I know, except Dr. Armstrong, who, in the epidemic at Sunderland, lost a smaller ratio than Dr. Rutter; whereas Gordon, Hey, Lee, Collins, and I believe every body else, lost patients under that epidemic and dreadful malady, in a much greater ratio to the recoveries. I have made these remarks because I am desirous to bear witness to the patience, the assiduity, the courage, the high intelligence, with which the amiable and worthy author of the letter bore himself, under the severe trial of his feelings throughout that whole epidemic constitution. I refer to the letter itself, and I recommend it to my medical brethren as a brilliant example of the success which is merited, and which is the best reward of a practice, founded on the incontrovertible doctrines of the pathology of puerperal metritis, phlebitis or peritonitis. Dr. Rutter's views need no riper comment than those which result from the relation he gives of this most interesting case. I shall offer none, but leave your readers to judge of it for themselves,

I am, sir, very respectfully,
Your obedient servant,
Ch. D. Meigs.

—

*Philadelphia, Dec.* 20, 1842.

Dear Sir,—Having again been so unfortunate as to meet with one of those horrible cases of puerperal fever, some of which you did me the favour to see with me during the past summer, I have thought that a short account of a recent case, with my treatment, might not be uninteresting to you.

Mrs. ——— was confined on Wednesday last, after a natural labour, with her third child, and continued well, with the exception of rather more severe after-pains than are usually felt, until 3 o'clock on Friday morning, when she was attacked with a severe chill, lasting more than one hour; violent fever succeeded, accompanied with excessive pain and tenderness, and with entire inability to move from the position in which she laid when the attack came on. I was called at 7 o'clock, the pulse was 136; lochia entirely suppressed; skin hot; countenance ghastly; tongue white and moist; the tenderness over the abdomen, and down the inside of the thighs, was extreme—indeed I have seldom seen at so early a period of the disease, so awful a case. I bled her 22 ounces by measure; gave 20 grains calomel with two grains of opium; stuping over the abdomen; cups to the sacrum. Ten o'clock, pulse 146; and every other symptom aggravated. I again bled her 20 ounces, when she fainted. After waiting a short time she recovered, with the pulse not any lessened in frequency. I again tied up the arm at 11 o'clock, and drew 10 ounces more; the pulse gradually lessened in frequency, though it appeared to lose none of its fulness. I now, finding that the calomel had not operated, gave her 20 grains more—with directions that it should be followed with a wine-glass full of a decoction of senna and manna every half hour. Two o'clock, pulse 90, soft; pain all gone, except when pressed upon. Six o'clock, pulse 84; medicine has operated very freely, producing very black and fetid stools, not unlike tar.

Ten o'clock, pulse 80; skin moist; but little pain or tenderness; gave her 10 grains of Dover's powder.

Saturday morning.—A slight return of pain and tenderness, but upon the whole pretty comfortable; can now change her position in bed. Evening; has slept well to day, and expresses herself as being free from pain or distress.

Sunday morning.—Has passed a comfortable night; milk is being secreted; pulse 84; the lochia fully established. The patient has continued to do well, has plenty of milk, and I do not detect an unfavourable symptom.

Tuesday evening.—The patient is still doing well, and I have no fear of the issue.

I have thus, my dear sir, endeavoured to give you a short account of this case, the result of which has been to me truly gratifying. Unfortunate as was the result of many of the cases I met with during the past year, I still flatter myself many others were saved by the prompt abstraction of blood, and from my experience in the disease, now numbering nearly 70 cases, all of which have occurred within less than a twelvemonth past, I am constrained to believe that the treatment as taught by yourself in Jefferson College, is that most to be relied upon.

As to the amount of blood which we may be privileged to take away, I would (with great caution and unwillingness to intrude an opinion) observe, we can scarcely err. If called within four hours after the attack, 18 or 20 ounces may be sufficient. If not called until eight hours have elapsed, that amount may have to be repeated; if not until 24 hours, we shall rarely be able to succeed without even a larger amount; if not until 36 hours have passed by, I fear that no treatment will save the patient. Purging with large and repeated doses of calomel,—assisted with some drastic article,—I hold to be all important. The other adjuvants in its treatment I consider of but little practical importance.

With sentiments of great respect,
I am yours, truly,
D. Rutter.

*Dr. Meigs.*

